# Inhibition of astrocyte BMP signaling alleviates neuroinflammation in experimental models of Parkinson’s disease

**DOI:** 10.1038/s41420-025-02812-2

**Published:** 2025-11-10

**Authors:** Yibo Li, Jiaxin Hao, Wenyu Wang, Zhaowen Su, Xiaofeng Tian, Hongfang Wang, Qing Liu, Jiamin Gao, Dandan Geng, Lei Wang

**Affiliations:** 1 Department of Human Anatomy, Hebei Medical University, Shijiazhuang, Hebei China; 2 Neuroscience Research Center, Hebei Medical University, Shijiazhuang, Hebei China; 3 Hebei Key Laboratory of Neurodegenerative Disease Mechanism, Shijiazhuang, Hebei China; 4 The Key Laboratory of Neural and Vascular Biology, Ministry of Education, Hebei Medical University, Shijiazhuang, Hebei China

**Keywords:** Astrocyte, Transcriptomics, Mechanisms of disease

## Abstract

Parkinson’s disease (PD) is clinically characterized by motor dysfunction, and its pathology primarily involves the progressive loss of dopaminergic neurons. Despite extensive research, the precise etiological mechanisms remain elusive. Recent findings have revealed a significant role of astrocytes in PD onset and progression. However, fully elucidating their function has been challenging because of the heterogeneity of cells and the complexity of the disease progression. Here, we successfully used single-nuclear RNA sequencing to characterize diverse genes expressed by astrocytes and analyzed changes in biological processes in a PD mouse model. Pseudotime analysis (Monocle2) indicated that as PD progressed the status of astrocytes transitioned from immune activation states to neurotoxic subtypes, which correlates with marked upregulation of inflammation-related genes, including Nlrp3 and IL-1β. Furthermore, CellChat analysis demonstrated that BMP signaling exhibited significant specificity in PD, with Bmp6 as the primary ligand and astrocytes as crucial mediators and responders. Thus, we demonstrated that BMP signaling activation in astrocytes exacerbates dopamine neuronal death. Rather, inhibiting BMP signaling in astrocytes significantly improved motor dysfunction and reduced the loss of dopamine neurons in a PD mouse model. Mechanistically, we found that activation of the BMP signaling pathway promoted the release of Nlrp3, IL-1β, and TNF-α, suggesting that increased neuroinflammation aggravated dopaminergic neuronal death. Together, these findings highlight the crucial role of astrocytes in the pathogenesis of PD and reveal a novel cellular mechanism that offers potential therapeutic targets for PD intervention.

## Introduction

Parkinson’s disease (PD) is a common neurodegenerative disorder characterized by significant clinical symptoms such as tremors, bradykinesia, rigidity, and gait abnormalities [1], alongside nonmotor symptoms [2], including depression, anxiety, and memory loss. The primary pathological features of this disease include the accumulation of alpha-synuclein (α-Syn) and the progressive loss of dopaminergic neurons [3].

The prevalence of PD significantly increases with age, affecting approximately 1–2% of individuals over 65 years of age, and this percentage increases to 4–5% among those over 85 years of age [4]. Currently, there is no complete cure for PD, which poses a significant public health challenge. Therefore, a deeper exploration of its pathogenic mechanisms is crucial for identifying potential therapeutic targets.

As the most abundant cell type in the central nervous system, astrocytes are critical in maintaining homeostasis and can release various proteins that support neuronal functions [5, 6]. Studies have shown that astrocyte dysfunction exacerbates the pathogenesis and progression of PD. For example, in PD mouse models, reduced expression of transmembrane protein 164 (TMEM164) in astrocytes leads to the loss of dopaminergic neurons and subsequent motor deficits [7]. Additionally, mutations in LRRK2 G2019S alter communication between astrocytes and neurons via extracellular vesicles, inducing neuronal atrophy [8]. The increased activity of MAOB within astrocytes enhances the synthesis and release of gamma-aminobutyric acid (GABA), subsequently inhibiting the activity of nearby dopaminergic neurons, which worsens motor symptoms [9]. However, the understanding of the specific roles of astrocytes in the progression of PD remains insufficient.

Recent advancements in single-cell/nuclear RNA sequencing technology (scRNA-seq or snRNA-seq) have provided valuable tools for elucidating the expression characteristics of different cell types under diseased conditions, which enables researchers to identify potential pathological states and the roles of the cell types in disease progression [10]. Previous studies have revealed that cell types show disease-specific transcriptional characteristics, such as oligodendrocytes observed in multiple sclerosis [11]^,^ and microglia identified in Alzheimer’s disease [12]. Recently, disease-associated astrocytes have also gained increasing attention [13].

In this study, we employed snRNA-seq to investigate the responses and gene expression changes in astrocytes in the substantia nigra (SN) of MPTP-induced subacute PD model mice [14]. We first performed gene set enrichment analysis (GSEA), Gene Ontology (GO) analysis, and Kyoto Encyclopedia of Genes and Genomes (KEGG) analysis to analyze the DEGs and their functions in astrocytes. We subsequently assessed the changes in astrocyte subclusters through pseudotime analysis and functionally annotated these subclusters. Finally, we utilized CellChat analysis to infer communication networks between astrocytes and other cell types, and revealed that astrocytes receive abundant PD-specific bone morphogenetic protein (BMP) signals. Further analysis indicated that inhibiting BMP signaling in astrocytes could reduce the expression of inflammation-related genes (such as Nlrp3, TNF-α, and IL-1β), thereby rescuing the loss of dopaminergic neurons and improving motor function in PD model mice. In summary, our study enhances the understanding of the heterogeneity of astrocytes in an MPTP-induced PD model and provides new insights into their roles in PD.

## Results

### Astrocytes DEGs and functional analysis in the MPTP mouse model

**Fig. 1:**
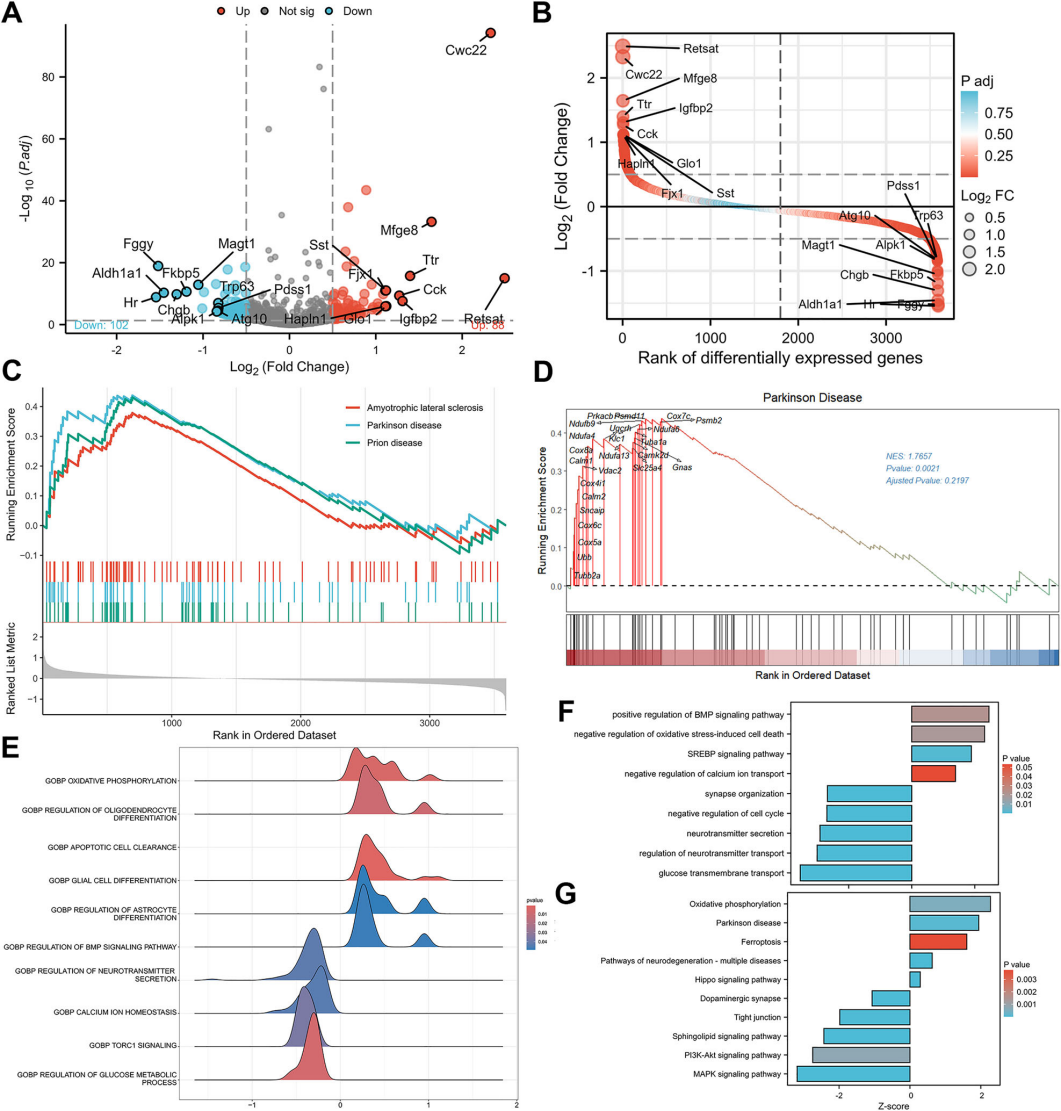
Astrocyte DEG analysis. **A** Volcano plot and **B** differential ranking plot of DEGs in astrocytes. The annotated genes were the top 10 upregulated and downregulated genes. **C** Visualization of GSEA results for DEGs in astrocytes. **D** GSEA results for the “Parkinson’s disease” pathway, with 19 annotated genes. **E** GSEA enrichment map of DEGs in astrocytes. **F** Bar plots of GO and **G** KEGG term enrichment for DEGs in astrocytes.

To investigate the role of astrocytes in PD, we analyzed astrocytes in mice based on our previous snRNA-seq data from the SN of mice treated with MPTP (PD) and control (CON) mice (Supplementary Fig. 1A–D) [14]. Differential gene expression analysis of astrocytes from PD mice compared with those from CON mice revealed 102 downregulated genes and 88 upregulated genes (*p* < 0.05, |LogFC| > 0.5) (Fig. 1A, B). To explore the biological processes related to these DEGs, GSEA was performed on the astrocyte DEG transcript lists via the Molecular Signatures Database (MSigDB) gene expression datasets. In the PD group, enrichment of pathways related to PD was observed (Fig. 1C), with 19 genes associated with this pathway (Fig. 1D). GSEA revealed that pathways such as “oxidative phosphorylation”, “glial cell differentiation”, and “regulation of the BMP signaling pathway” were activated in PD mice. In contrast, processes such as “regulation of neurotransmitter secretion”, “calcium ion homeostasis”, and “regulation of glucose metabolism processes” were inhibited in PD mice compared with CON mice (Fig. 1E).

Performed using clusterProfiler (for Gene Ontology [GO] and Kyoto Encyclopedia of Genes and Genomes [KEGG] enrichment analysis) and GSEA revealed similar functional enrichment patterns. GO functional annotation revealed upregulated pathways, including “positive regulation of the BMP signaling pathway”, “negative regulation of oxidative stress-induced cell death”, “SREBP signaling pathway”, and “negative regulation of calcium ion transport” (Fig. 1F). KEGG analysis revealed enrichment of pathways such as “oxidative phosphorylation”, “Parkinson’s disease”, “ferroptosis”, and “Hippo signaling pathway” (Fig. 1G). Furthermore, GO functional annotation revealed that the pathways inhibited in PD include the secretion and transport of neurotransmitters, which is consistent with the GSEA results.

In conclusion, these analyses elucidate the biological processes and associated pathways of astrocytes based on snRNA-seq data, thereby advancing our understanding of the regulatory mechanisms governing their function in PD.

### Subpopulation reconstruction of astrocytes

**Fig. 2:**
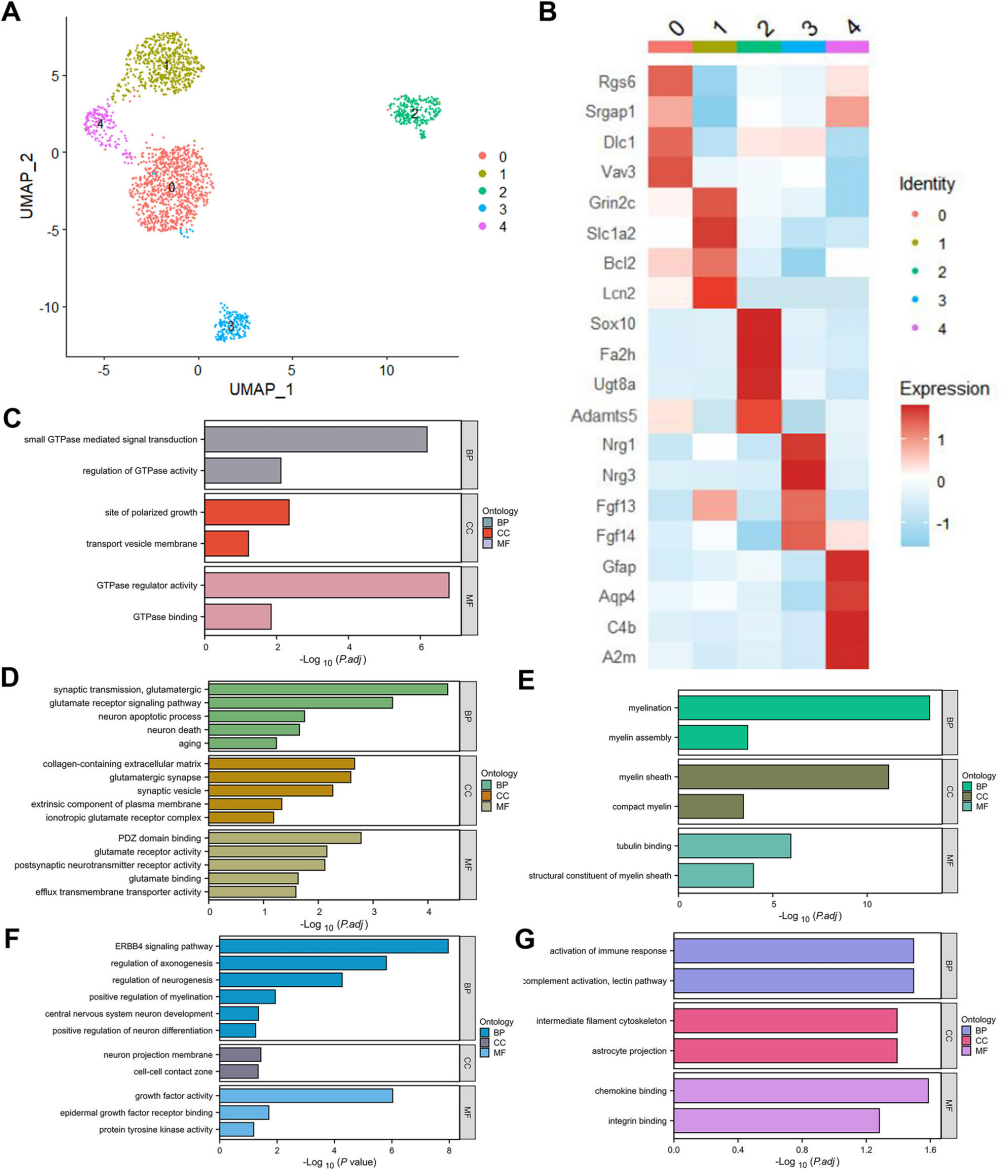
Astrocyte subpopulation reconstruction. **A** UMAP embedding of astrocyte nuclei, colored by cluster. **B** Heatmap of marker gene expression in astrocyte clusters. **C**–**G** Bar plots of GO functional annotations for marker gene expression in astrocyte clusters 0–4.

To further investigate the heterogeneity of the astrocyte cluster, we reclustered the astrocyte nuclei, identifying 5 distinct subclusters (clusters 0–4) (Fig. 2A). Marker genes characteristic of each subcluster were then identified based on their gene expression profiles (Fig. 2B). These marker genes were subsequently analyzed for functional enrichment using GO analysis.

Cluster 0 presented high expression levels of Rgs6, Srgap1, Dlc1, and Vav3 (Fig. 2B). GO analysis revealed significant enrichment in small GTPase-mediated signal transduction, regulation of GTPase activity, and transport vesicle membrane functions within this cluster (Fig. 2C). Additionally, we determined that Grin2c, Slc1a2, Bcl2, and Lcn2 were highly expressed in cluster 1 (Fig. 2B). GO analysis revealed that astrocytes in cluster 1 are associated with functions related to the glutamate receptor signaling pathway, neuronal apoptosis, and neuronal death (Fig. 2D). Cluster 2 presented high expression levels of Sox10, Fa2h, Ugt8a, and Adamts5 (Fig. 2B), with functions enriched primarily in myelin sheath-related processes (Fig. 2E). Previous studies have shown that astrocytes are crucial in regulating demyelination and remyelination [15] and are essential for maintaining normal myelin sheath integrity [16]. Cluster 3 exhibited elevated expression levels of Nrg1, Nrg3, Fgf13, and Fgf14 (Fig. 2B), with functions associated with promoting neuronal survival, neurogenesis, and differentiation (Fig. 2F). Finally, cluster 4 presented increased expression levels of Gfap, Aqp4, C4b, and A2m (Fig. 2B). GO analysis revealed that the molecular functions enriched in cluster 4 predominantly involved the activation of the immune response, complement activation, and astrocyte projections (Fig. 2G). Based on this analysis, we conclude that different astrocyte subclusters exhibit distinct activation states.

### Trajectory analysis of astrocytes

**Fig. 3:**
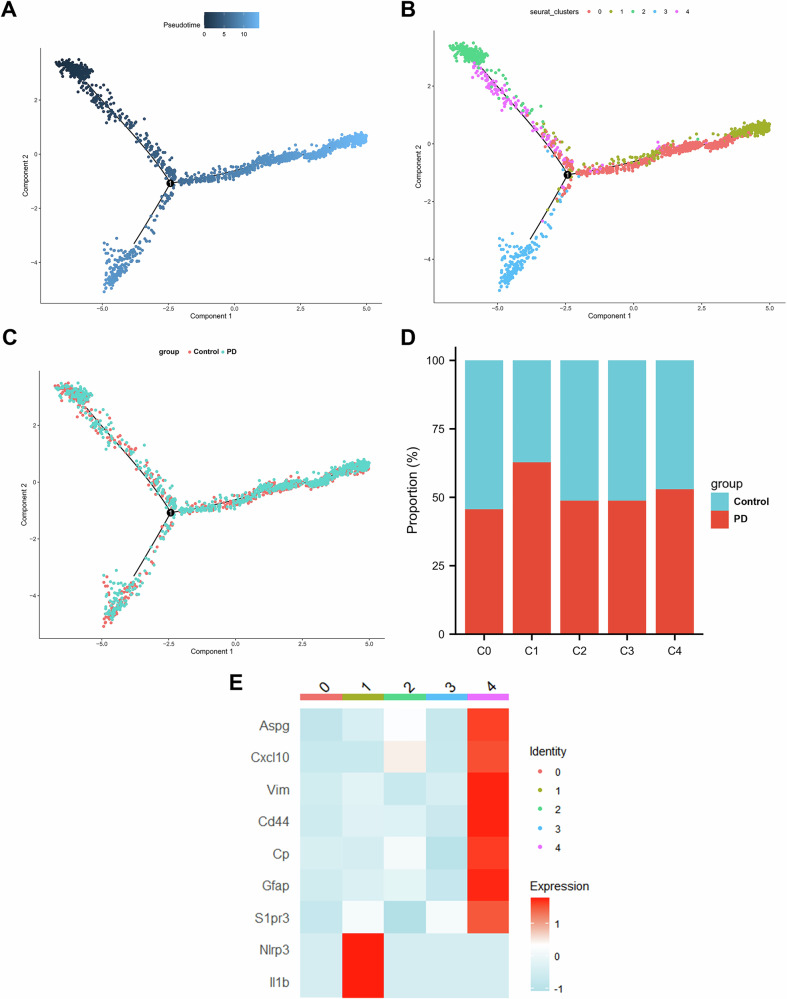
Astrocyte trajectory analysis. **A** Pseudotime analysis using Monocle2 was performed to visualize the metastatic trajectory of distinct cell clusters. The color gradient indicates progression along pseudotime. **B** The positions of each astrocyte subtype on the pseudotime trajectory are shown, with each cluster marked by a different color. **C** The metastatic trajectories of the control and PD groups are displayed. **D** Proportion of control and PD cells in each cluster. **E** Heatmap showing the characteristic genes of panreactive astrocytes in cluster 4 and inflammation-related genes in cluster 1.

We subsequently performed pseudotime analysis with Monocle2 to reconstruct the activation trajectory and elucidated the transitional relationships among identified subclusters. Adamts5 is a metalloproteinase involved in cell development and cell migration and is upregulated during astrocyte development [17]. Compared with the other clusters, Cluster 2 specifically expressed Adamts5 (Fig. 2B), so we considered Cluster 2 as the least mature branch in pseudotime (Fig. 3A, B). This astrocyte activation trajectory spans from cluster 2 toward two activation branches, one containing cluster 3 and another with cluster 1 highly expressing Lcn2 and Bcl2. Lcn2 was recently reported to trigger neuronal death and to contribute to astrocyte toxicity [18]. Moreover, the proportion of astrocytes in cluster 1 was significantly greater in the PD group compared to the CON group (Fig. 3C, D). From this, we estimated that the activation trajectory of astrocytes spans from cluster 2 toward cluster 1. The astrocytes in the “transitional state” cluster were in cluster 4 and cluster 0, which showed enrichment for the immune response and positive regulation of GTPase activity (Fig. 2C, J). Recent studies have shown that the upregulation of signals mediated by GTPases promotes astrocyte polarization into the neurotoxic subtype [19]. The activation of the immune response in astrocytes promotes neuroinflammation through various pathways, including the release of cytokines, production of chemokines, modulation of microglia, and interaction with peripheral immune cells [20]. Cluster 4 was characterized by specific expression of Gfap, which is characteristic of reactive astrocytes. Nlrp3 and IL-1β inflammation-related genes were significantly increased in the terminal neurotoxic astrocyte population (cluster 1) (Fig. 3E). Therefore, we hypothesize that the activation of the astrocytic complement pathway and immune response contribute to astrocyte-induced toxicity in PD. This mechanism likely enables astrocytes to release of pro-inflammatory cytokines, ultimately leading to neuronal damage and cell death [21]. In summary, pseudotime analysis successfully revealed the dynamic transitions of astrocyte subclusters and highlighted a trajectory associated with reactive and potentially neurotoxic phenotypes during PD progression.

### Cell-cell communication analysis revealed specific activation of the BMP signaling pathway

**Fig. 4:**
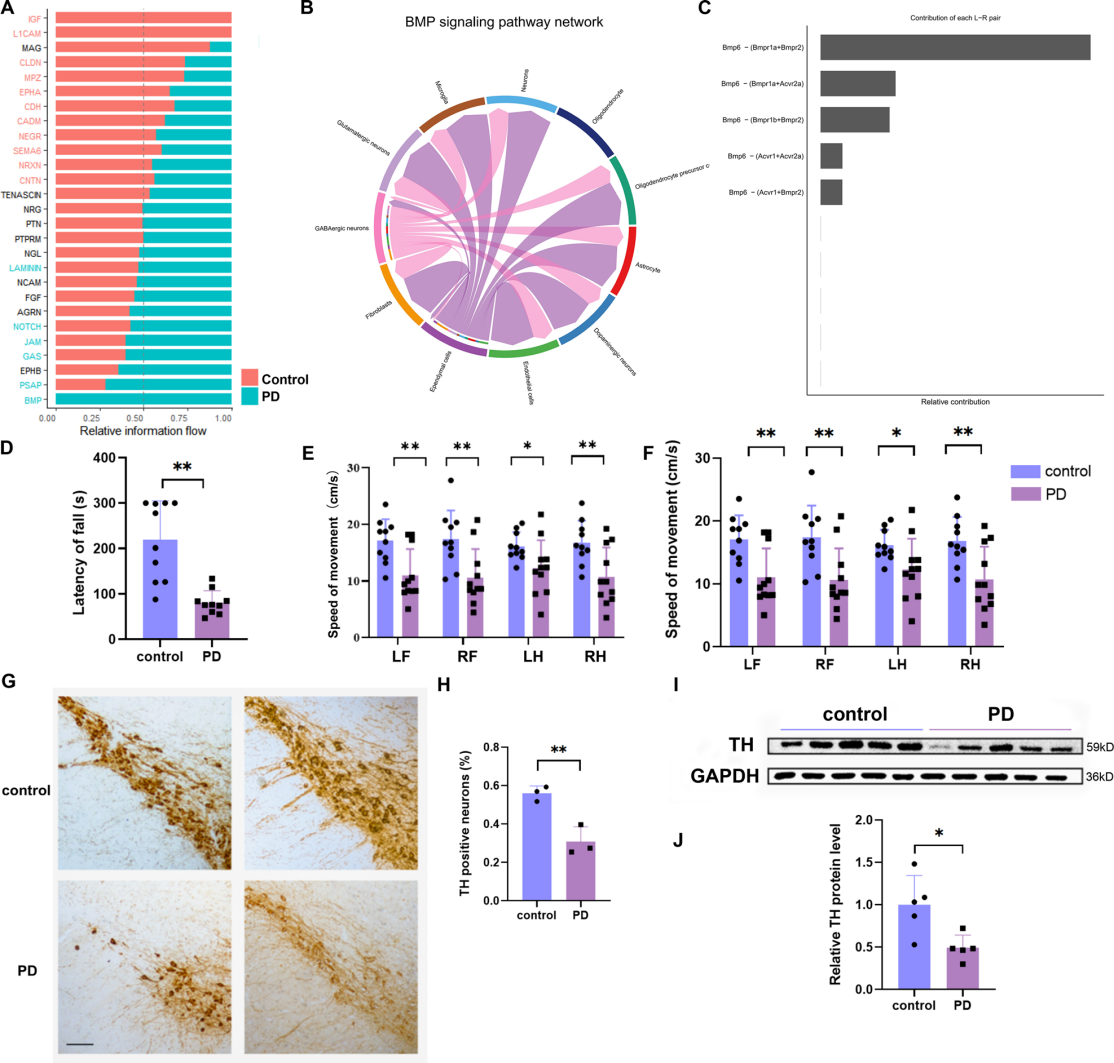
Cell-cell communication analysis revealed specific activation of the BMP signaling pathway. **A** Signaling pathways were ranked by differential information flow (total communication probability) between PD and control networks. Pathways enriched in the control group exhibited higher information flow, while those enriched in the PD group exhibited lower information flow. **B** The inferred BMP signaling network. **C** Relative contribution of each L-R pair to the overall BMP signaling network at PD. **D**–**F** Behavioral results of 3-month-old C57BL/6 mice following saline (Con) or MPTP (PD) injection: **D** Rotarod test; **E**, **F** Gait analysis via the WalkAnalysator system. *n* = 10 mice per group. **G** Representative images and (**H**) quantification of TH^+^ neurons in the Substantia Nigra pars compacta (SNpc) for each group. Scale bar = 100 μm, *n* = 3. **I** Western blot (WB) analysis and **J** quantification of TH protein levels in the SNpc, *n* = 5. Data are shown as the mean ± SEM. *p-*values, two-tailed *t*-test, *p < 0.05, **p < 0.01.

Building on these findings, the biological functions of astrocytes in PD mice were found to be altered, encompassing processes such as “neuroinflammation”, “neuronal apoptosis process” and “neuronal death”. To gain a deeper understanding of astrocyte function within their broader cellular environment, we employed CellChat to infer the changes in communication among all cell types. This approach clarifies how astrocytes respond to signal variations from other cell types. The CellChat results revealed that “BMP signaling pathways” were specifically enriched in the PD group (Fig. 4A). Figure 4B visualizes the incoming and outgoing BMP signals among various cell types. Furthermore, we examined previously reported human midbrain scRNA-seq data [22]. Consistent with our findings in the mouse model, the BMP signaling pathway was also found to be PD-specific in humans (Supplementary Fig. 2A). In contrast to the mouse data, the BMP signaling pathway was specifically activated and primarily mediated by the ligands BMP6 and BMP7 (Supplementary Fig. 2B).

BMPs belong to the transforming growth factor (TGF)-β superfamily of signaling proteins. More than 12 BMP family proteins are found in most mammals, including BMP2, BMP4, BMP5, BMP6, BMP7, BMP8, BMP9, and BMP10 [23]. Our sequencing results revealed Bmp6 as the primary ligand involved in the BMP signaling pathway (Fig. 4C). The activation of canonical BMP signaling by BMPs has been extensively reviewed [24, 25]. Briefly, BMP ligands bind to a type II receptor, which in turn associates with and phosphorylates a type I receptor. The type I receptor then recruits and phosphorylates receptor-regulated Smads (R-Smads), typically Smad1, Smad5, and Smad8 (also referred to as Smad1/5/9 or Smad1/5/8). These phosphorylated R-Smads form a complex with the cofactor Smad4 that translocates into the nucleus to directly regulate the transcription of target genes [26]. Id1, Id2, Id3, and Id4 are direct targets of phosphorylated Smads (*p*-Smad) in the canonical BMP signaling pathway, and their expression is upregulated upon BMP pathway stimulation [27].

**Fig. 5:**
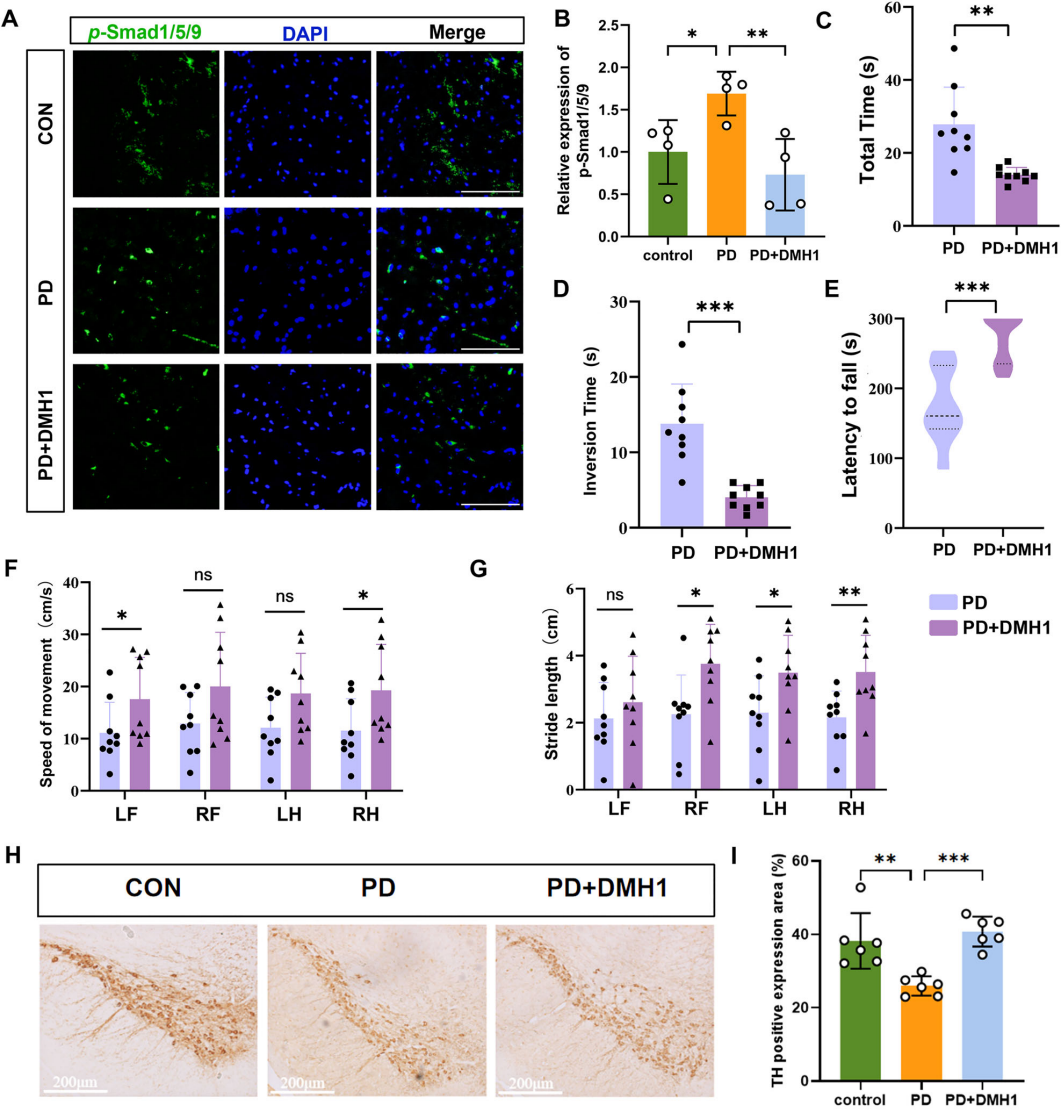
BMP signaling pathway was activated in the MPTP mouse model. **A**, **B** Immunostaining data showing *p*-Smad1/5/9 intensity in the SN of Con and PD group mice. Scale bar = 100 μm, *n* = 4. **C**–**G** Behavioral results of 3-month-old C57BL/6 mice following saline (Con) or MPTP (PD) injection: **C**, **D** Climbing pole test; **E** Rotarod test; **F**, **G** Gait analysis via the WalkAnalysator system. *n* = 9 mice per group. LF left front foot, RF right front foot, LH left rear foot, RH right rear foot. **H** Representative images and **I** quantification of TH^+^ neurons in the SNpc for each group. Scale bar = 200 μm, *n* = 6. Data are shown as the mean ± SEM. *p-*values, two-tailed *t*-test, one-way ANOVA. *p < 0.05, **p < 0.01, ***p < 0.001.

The subacute PD mouse model were generated by intraperitoneal injection of MPTP, and behavioral experiments demonstrated a significant reduction in motor function following MPTP administration (Fig. 4D–F). Both immunohistochemical staining (Fig. 4G, H) and western blot analyses (Fig. 4I, J) confirmed a significant decrease in tyrosine hydroxylase (TH) expression of PD model mice compared to control (CON) mice. This validated the successful establishment of the PD model and indicated dopaminergic neuronal damage. Subsequently, SN tissue sections from PD and CON mice were subjected to immunofluorescence staining. The results showed significantly increased phosphorylation of Smad1/5/9 in the SN of the PD group compared to the CON group. To investigate the role of BMP signaling, we inhibited this pathway using DMH1, a known inhibitor of activin receptor-like kinase 2 (ALK2) [28]. As a type I receptor, ALK2 is involved in BMP6 signaling and mediates the phosphorylation of Smad1/5/9 [29]. Immunofluorescence staining confirmed that DMH1 treatment significantly reduced the phosphorylation of *p*-Smad1/5/9 in the SN of PD mice (Fig. 5A, B). These results collectively indicated that the BMP signaling pathway was significantly activated in the PD group.

Furthermore, we investigated whether inhibiting the BMP signaling pathway could ameliorate motor function in PD mice. The climbing pole test showed significantly reduced the total pole climbing time and head turning time in the PD + DHM1 group compared to the PD group (Fig. 5C, D). The rotarod test demonstrated significantly increased the fall latency in the PD + DHM1 group compared to the PD group (Fig. 5E). Gait analysis further revealed significant increases in stride length and step speed in the PD + DMH1 group compared to the PD group (Fig. 5F, G). Immunohistochemical staining showed significantly higher TH expression in the SN of the PD + DMH1 group compared to the PD group (Fig. 5H, I). These results collectively indicate that inhibiting the BMP signaling pathway alleviated dopaminergic neuron loss and motor function in the PD model.

### Astrocyte BMP signaling pathway was activated in the MPTP mouse model

**Fig. 6:**
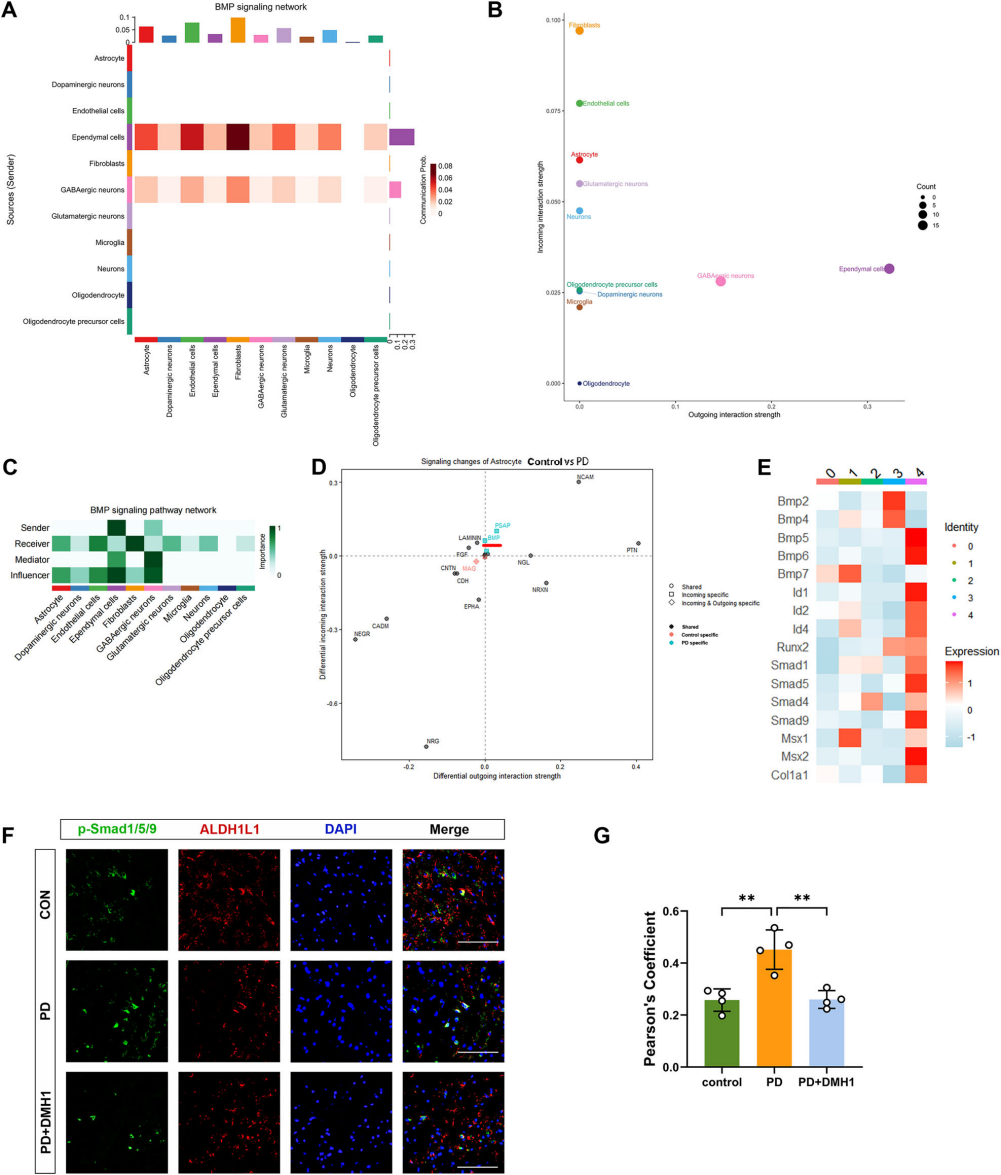
The astrocyte BMP signaling pathway was activated in the MPTP mouse model. **A** Heatmap and **B** bubble plot of the interactions between the BMP signaling pathway sending and receiving cells. In addition to fibroblasts and endothelial cells, astrocytes are also key cell types that receive BMP signals. **C** Heatmap showing the network centrality index for each cell type. **D** Subsequent differential analysis of the interaction strength between astrocytic input and output signals. **E** Heatmap showing the specific expression of BMP signaling-related genes in “transition state” Cluster 4. **F**, **G** Immunostaining data showing *p*-Smad1/5/9 intensity in Aldh1l1^+^ astrocytes in the SN of Con and PD group mice. Scale bar = 100 μm, *n* = 4. Data are shown as the mean ± SEM. *p*-values, one-way ANOVA. **p < 0.01.

In the CellChat heatmaps, the vertical axis represents the sender cells, while the horizontal axis represents the receiver cells. The color intensity indicates the signal strength, and the upper and right margins display the cumulative outgoing and incoming signal strength for each cell type, respectively. Within the BMP signaling pathway heatmap, in addition to fibroblasts and endothelial cells, the upper margin related to astrocytes presented the greatest cumulative incoming signal strength (Fig. 6A). A bubble plot visualizing the incoming and outgoing BMP signal strength for each cell type (Fig. 6B) further demonstrated that astrocytes received BMP signals with an intensity second only to that of fibroblasts and endothelial cells. By calculating the network centrality indices for each cell population, we confirmed the roles (i.e., sender, receiver, mediator, and influencer) of each cell type, highlighting that astrocytes function as significant receivers and influencers in the BMP signaling network (Fig. 6C). Consistent with these findings in the mouse model, analysis of the human dataset (Supplementary Fig. 2C, D) also identified astrocytes as major receivers and influencers in the BMP signaling pathway. Subsequent differential analysis of the interaction strength of astrocytic input and output signals revealed a significant increase in BMP signal reception (Fig. 6D). Furthermore, we observed a significant increase in the expression of Id1, Id2, and Id4 within the “transition state” cluster (cluster 4). Id1, Id2, Id3, and Id4 are direct targets of *p*-Smad in the canonical BMP signaling pathway [27], these findings indicate specific activation of BMP signaling within this subset of astrocytes in the PD model (Fig. 6E).

To further confirm that the astrocyte BMP signaling pathway was activated in PD, we evaluated the expression of *p*-Smad1/5/9 in astrocytes through double immunofluorescence staining. Qualitative analysis revealed a marked increase in *p*-Smad1/5/9 levels in Aldh1l1-positive astrocytes in the PD group compared with those in the CON group (Fig. 6F, G). Furthermore, administration of the inhibitor DMH1 to PD mice significantly reduced *p*-Smad1/5/9 levels in Aldh1l1-positive astrocytes compared to untreated PD mice (Fig. 6F, G).

**Fig. 7:**
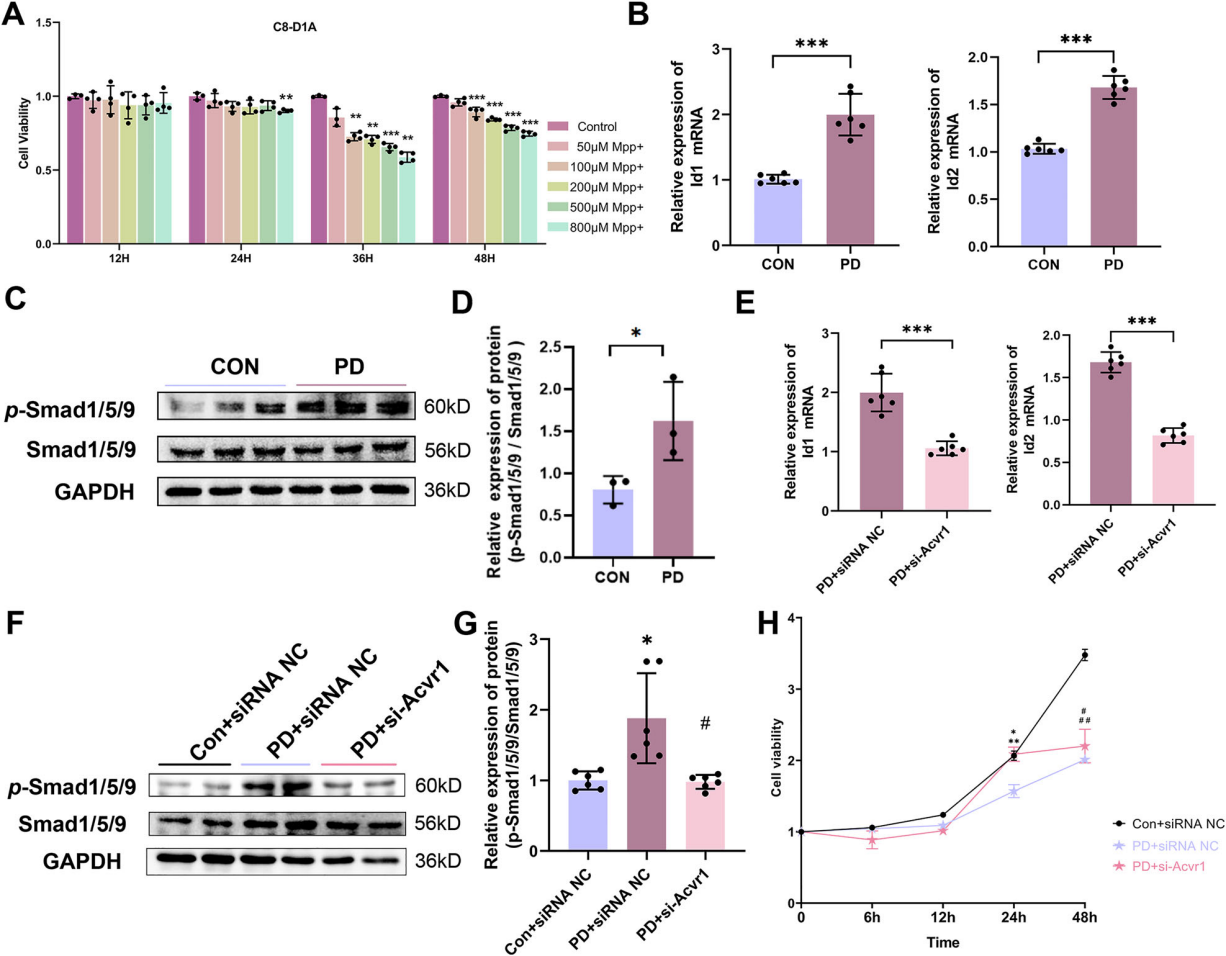
BMP signaling pathway activation in the MPP^+^-treated astrocyte model. **A** CCK-8 assay showing the viability of MPP^+^-treated astrocytes (C8-D1A). **B** qRT-PCR analysis of the mRNA expression of the BMP signaling downstream target genes Id1 and Id2. **C** WB analysis and **D** quantification of *p*-Smad1/5/9 protein levels in MPP^+^-treated astrocytes. **E** qRT-PCR analysis of Id1 and Id2 mRNA expression in astrocytes treated with MPP^+^ and transfected with siRNA to inhibit BMP signaling. **F** WB analysis and **G** quantification of *p*-Smad1/5/9 protein levels in astrocytes treated with MPP^+^ and transfected with siRNA to inhibit BMP signaling. **H** CCK-8 assay showing the viability of astrocytes treated with MPP^+^ and transfected with siRNA to inhibit BMP signaling. *p < 0.05 vs. the Con+siRNA NC group; *#p* < 0.05 vs. the PD+siRNA NC group; the data are presented as the means ± SEMs. *p*-values, one-way ANOVA.

Next, we applied MPP^+^ treatment to C8-D1A astrocyte cell line to simulate the PD environment. The CCK-8 assay was utilized to identify the optimal conditions for treating astrocytes with MPP^+^. We observed a significant reduction in the proliferation rate of astrocytes at a concentration of 800 µM after 24 h (Fig. 7A). Under these optimal conditions, MPP^+^ markedly activated the classical BMP target genes Id1 and Id2 (Fig. 7B) and increased *p*-Smad1/5/9 levels (Fig. 7C, D), suggesting significant activation of BMP signaling in astrocytes. To inhibit the BMP signaling pathway in astrocytes, we designed a siRNA targeting the ALK2 receptor (si-Acvr1). After the astrocytes were transfected with si-Acvr1, the cells were treated with MPP^+^ under the same conditions. In the MPP^+^ combined with si-Acvr1 group (PD + si-Acvr1 group), the expression of Id1 and Id2 was significantly reduced (Fig. 7E), and a notable decrease in *p*-Smad1/5/9 levels was observed (Fig. 7F) compared with those in the MPP^+^ combined with siRNA negative control group (PD + siRNA NC group). Additionally, the CCK-8 assay revealed an increased proliferation rate of astrocytes in the PD + si-Acvr1 group (Fig. 7H).

### Activation of the BMP signaling pathway leads to the release of pro-inflammatory cytokines

Given the observed protective effect of inhibiting BMP signaling on astrocytes in the PD model, we investigated the mechanisms by which BMP signaling alters astrocyte function. The sequencing results revealed a significant increase in the expression of inflammation-related genes, including Nlrp3 and IL-1β, within the terminally toxic astrocyte cluster (Cluster 1) (Fig. 2D). Furthermore, the BMP signaling pathway was activated in the “transitional astrocytes” cluster (Cluster 4) (Figs. 3B and 6E). Collectively, these findings suggest that the activation of the BMP signaling in astrocytes may contribute to neuroinflammation.

**Fig. 8:**
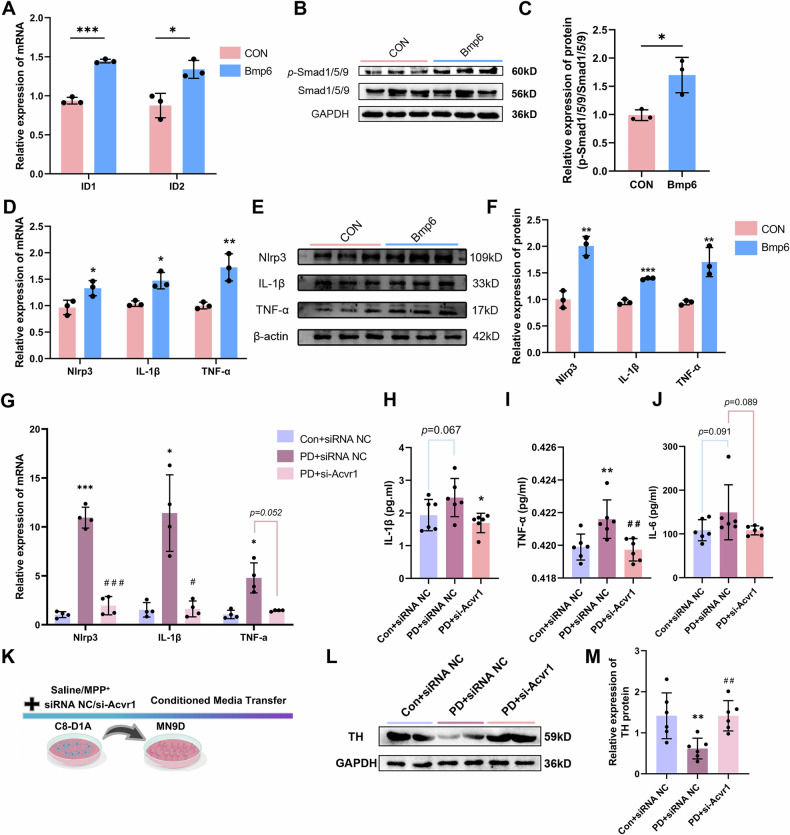
The activation of the BMP signaling pathway leads to the release of pro-inflammatory cytokines. Exogenous recombinant Bmp6 (100 ng/ml) was used to enhance BMP signaling in astrocytes (C8-D1A) for expression level analysis. **A** qRT-PCR analysis of the mRNA expression of Id1 and Id2. **B** WB analysis and **C** quantification of *p*-Smad1/5/9 protein levels. **D** qRT-PCR analysis of the mRNA expression of Nlrp3, IL-1β, and TNF-a. **E** WB analysis and **F** quantification of Nlrp3, IL-1β, and TNF-a protein levels (*n* = 4). Astrocytes were treated with MPP^+^, and the expression levels were measured after BMP signaling inhibition via siRNA NC or si-Acvr1. **G** qRT-PCR analysis of the mRNA expression of Nlrp3, IL-1β, and TNF-a (*n* = 4). **H–J** ELISA analysis of **H** IL-1β, **I** TNF-α and **J** IL-6 protein levels in the supernatant of astrocytes after treatment (*n* = 6). **K** Astrocytes were treated with MPP^+^ and simultaneously transfected with siRNA NC or si-Acvr1, and the collected medium was used for conditioned culture of dopamine neurons (MN9D). **L** WB analysis and **M** quantification of TH protein levels in dopamine neurons (*n* = 6). *p < 0.05, **p < 0.01, ***p < 0.001 vs. the Con+siRNA NC group; ^*#*^*p* < 0.05, ^*##*^*p* < 0.01, ^*###*^*p* < 0.001 vs. the PD + siRNA NC group. Data are presented as the means ± SEMs. *p*-values, one-way ANOVA.

We subsequently treated astrocytes with exogenous recombinant Bmp6 (100 ng/ml) to activate BMP signaling in astrocytes and observed the consequent release of inflammatory factors. Treatment significantly increased the expression of classical BMP target genes Id1 and Id2 (Fig. 8A) and elevated *p*-Smad1/5/9 levels (Fig. 8B, C), confirming successful activation of the BMP signaling. As anticipated, the expression of inflammation-related genes, including Nlrp3, TNF-α, and IL-1β, at both mRNA and protein levels was significantly elevated (Fig. 8D–F).

Furthermore, an in vitro model of astrocytes was induced via MPP^+^, and the expression of relevant inflammatory factors was assessed through Western blot and qRT-PCR. The expression levels of Nlrp3, TNF-α, and IL-1β were significantly elevated in the PD + siRNA NC group compared with those in the CON + siRNA NC group, whereas they were decreased in the PD + si-Acvr1 group relative to those in the PD + siRNA NC group (Fig. 8G). The levels of IL-6, TNF-α, and IL-1β in the culture supernatant were measured by ELISA, and consistent results were obtained (Fig. 8H–J). These findings suggest that the activation of astrocytic BMP signaling enhances the release of pro-inflammatory cytokines.

### Inhibiting astrocyte BMP signaling improved motor function in MPTP-treated mice

To investigate the impact of the astrocytic BMP signaling pathway on dopaminergic neurons, we established three experimental groups for astrocytes: CON + siRNA NC, PD + siRNA NC, and PD + si-Acvr1. Conditioned medium from these groups was then collected and used to culture dopamine neurons (MN9D) (Fig. 8K). Relative to neurons cultured in medium from the CON + siRNA NC group, the protein expression of TH was significantly lower in neurons cultured in medium from the PD + siRNA NC group (Fig. 8L–M). In contrast, treatment with conditioned medium from the PD + si-Acvr1 group resulted in significantly increased levels of both TH mRNA and protein levels (Fig. 8L–M). These findings indicate that the inhibiting astrocyte BMP signaling exerts a protective effect on dopaminergic neurons.

**Fig. 9:**
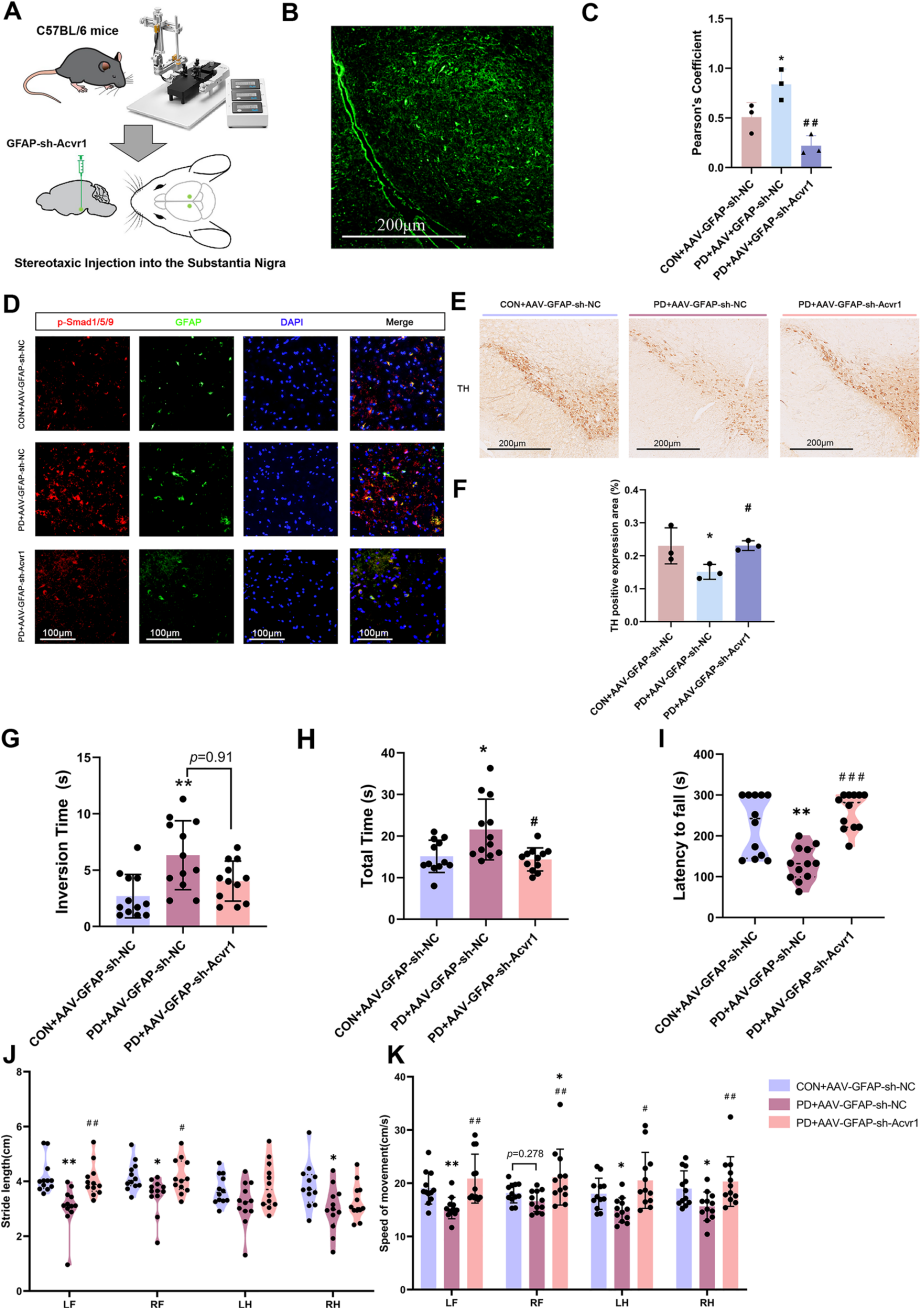
Inhibiting astrocyte BMP signaling improved motor function in MPTP-treated mice. **A** AAV was administered to the SN of mice to knock down the astrocyte-specific ALK2 receptor (GFAP-sh-Acvr1). **B** Mice injected with AAV5 carrying GFP under the astrocyte-specific promoter (GFAP) in the SN were verified to express GFP specifically in astrocytes. **C**, **D** Immunostaining data showing *p*-Smad1/5/9 intensity in Gfap^+^ astrocytes in the SN of Con and PD group mice. Scale bar = 100 μm, (*n* = 3). **E** Representative images and **F** quantification of TH^+^ neurons in the SNpc for each group. Scale bar = 200 μm, (*n* = 3). **G**–**K** Behavioral results of 3-month-old C57BL/6 mice following stereotaxic injection of AAV and subsequent injection of either saline (Con+AAV-GFAP-sh-NC) or MPTP (PD + AAV-GFAP-sh-NC/PD + AAV-GFAP-sh-Acvr1): **G**, **H** Climbing pole test; **I** Rotarod test; **J**, **K** gait analysis via the WalkAnalysator system. *n* = 12 mice per group. LF left front foot, RF right front foot, LH left rear foot, RH right rear foot. *p < 0.05, **p < 0.01, ***p < 0.001 vs. the CON + AAV-GFAP-sh-NC group; ^*#*^*p* < 0.05, ^*##*^*p* < 0.01, ^*###*^*p* < 0.001 vs. the PD + AAV-GFAP-sh-NC group; the data are shown as the means ± SEMs. *p*-values, one-way ANOVA.

To specifically inhibit astrocytic BMP signaling in vivo, we injected an adeno-associated virus (AAV) expressing shRNA targeting the ALK2 receptor (Acvr1) under the astrocyte-specific GFAP promoter (GFAP-sh-Acvr1) (Fig. 9A). We first verified that mice injected with AAV5 carrying green fluorescent protein (GFP) under the astrocyte promoter into the SN expressed GFP strictly in astrocytes (Fig. 9B). Immunofluorescence staining demonstrated that GFAP-sh-Acvr1 significantly reduced *p*-Smad1/5/9 levels in astrocytes (Fig. 9C, D). Relative to the PD + GFAP-sh-NC group, TH expression was significantly increased in the PD + GFAP-sh-Acvr1 group (Fig. 9E, F). Compared with those of PD model mice, the climbing pole test results revealed that PD + GFAP-sh-Acvr1 group significantly decreased the total climbing time and turning around time (Fig. 9G, H). Additionally, the rotarod test showed that the fall latency was significantly greater in the PD + GFAP-sh-Acvr1 group compared to the PD + GFAP-sh-NC group (Fig. 9I). Gait analysis further demonstrated a significant increase in step length and step speed in the PD + GFAP-sh-Acvr1 group compared to the PD + GFAP-sh-NC group (Fig. 9J, K). Collectively, these results suggest that inhibiting BMP signaling in astrocytes effectively rescued motor function in MPTP model mice.

## Discussion

**Fig. 10:**
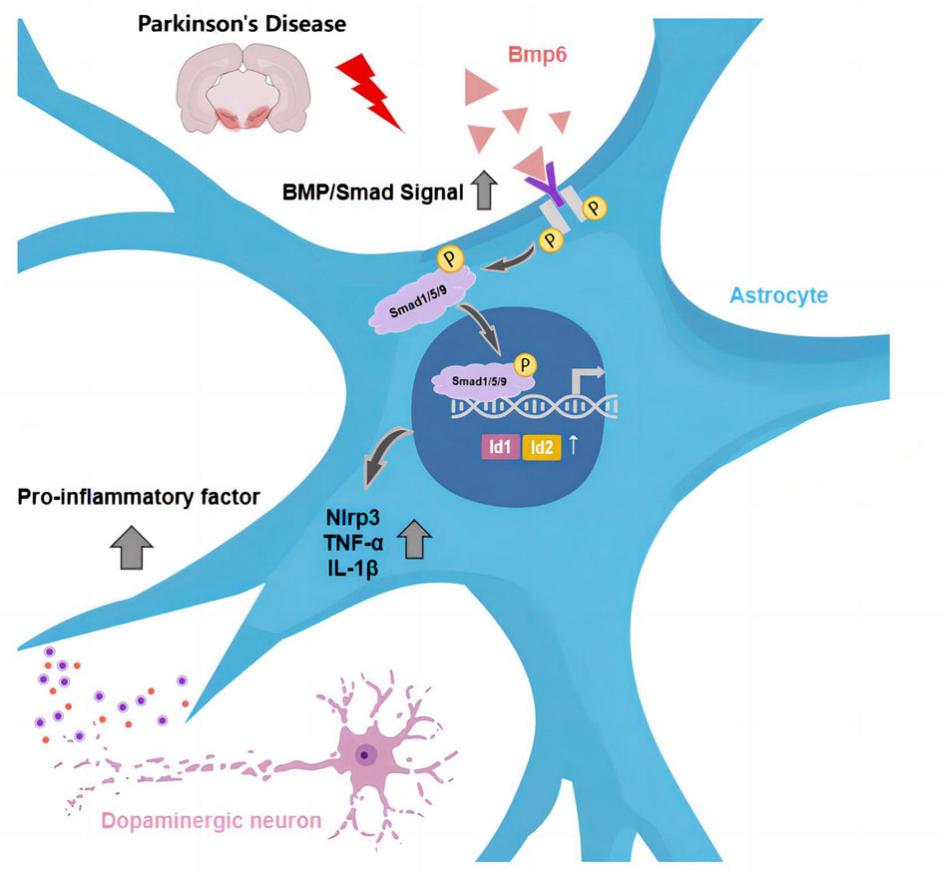
Schematic representation of the molecular mechanisms involved. Inhibition of BMP signaling in astrocytes reduces neuroinflammation and improves motor function and dopamine neuronal loss in PD model mice. Created with BioRender.com.

Our study significantly advances the understanding of the role of astrocytes in PD. For the first time, we associated the activation of the BMP signaling pathway in PD with astrocyte-mediated neuroinflammation. In our study, snRNA-seq revealed substantial molecular heterogeneity and dynamic transitions among distinct astrocyte subclusters. CellChat further indicated that astrocytes act as important recipients and regulators of the BMP signaling pathway. Importantly, specific inhibition of BMP signaling in astrocytes greatly reduced the expression of key pro-inflammatory genes (Nlrp3, TNF-α, and IL-1β), which led to improved motor function and reduced dopamine neuronal loss in PD model mice (Fig. 10).

Prior studies have highlighted the significant role of astrocytes in PD pathogenesis. For example, impaired oxidative phosphorylation in astrocytes has been shown to compromise their support for dopaminergic neuron survival, leading to neuronal loss [30]. Blocking the differentiation of C3+ neurotoxic astrocytes reduces dopaminergic neuronal loss and alleviates behavioral deficits in preclinical PD models [31]. Astrocytes also synthesize and release neurotransmitters such as glutamate and γ-aminobutyric acid. The compromised functionality of astrocytes can lead to decreased synthesis and release of these neurotransmitters in PD [32, 33]. In line with these findings, our detailed snRNA-seq analysis of the SN from MPTP-induced subacute PD model mice further investigated the multifaceted roles of astrocytes. Our findings indicate that DEGs in the astrocytes were enriched in pathways including oxidative phosphorylation, glial differentiation, and BMP signaling. Conversely, pathways associated with neurotransmitter secretion and calcium ion homeostasis were found to be downregulated. These findings collectively underscore the complex molecular reprogramming of astrocytes in PD, affecting processes such as glucose metabolism and neurotransmitter secretion, and provide further support for the growing evidence of the critical role of astrocytes in neurodegenerative disorders [34, 35].

To visualize continuous changes in astrocyte profiles during PD progression, pseudotime analysis was employed to align cells along trajectories from cluster 2 and to cluster 1. This analysis revealed that astrocytes in the “transitional state” presented increased activation of the immune response. Furthermore, the terminal neurotoxic astrocyte population (cluster 1) showed significant increase in the expression of key pro-inflammatory genes, including Nlrp3 and IL-1β. In neurodegenerative diseases, astrocytes are dysregulated in pathological states, which can induce neuroinflammation [36, 37]. Integrating these findings with existing knowledge, we hypothesize that the activation of the astrocytic immune response contributes to astrocyte-induced toxicity in PD. This mechanism enables astrocytes to drive neuroinflammation by releasing elevated levels of pro-inflammatory cytokines, ultimately leading to neuronal damage and cell death [21].

Utilizing CellChat analysis, we explored how astrocyte functions are altered in PD mice and how astrocytes respond to signals from other cell types. Our study presents compelling evidence from both CellChat analyses and immunofluorescence staining, indicating that the BMP signaling pathway is specifically activated in PD, with astrocytes recognized as crucial mediators in this process. To bolster the translational relevance of these findings, we analyzed publicly available human PD snRNA-seq data, which confirmed similar BMP signaling activation, further supporting the clinical potential of targeting BMP signaling in astrocytes as a therapeutic strategy for PD. The BMP signaling pathway plays a critical role in regulating multiple biological processes in glial cells. In oligodendrocyte precursor cells, activation of BMP signaling inhibits myelination; conversely, in demyelinating lesions associated with multiple sclerosis, inhibition of this pathway can promote myelin regeneration [38]. In microglia, BMP signaling activation, through the regulation of IL-6, affects HAMP gene expression and hepcidin secretion, which participate in the regulation of iron homeostasis [39]. In astrocytes, BMP9 overexpression significantly reduces cell death and enhances viability by promoting ERK activation, thereby providing protective effects during brain ischemia-reperfusion [40]. The classical BMP signaling pathway is pivotal in regulating the expression and subcellular localization of AQP4 in astrocytes, thereby maintaining homeostasis within the brain environment [41, 42].

Although prior research highlights the importance of BMP signaling in astrocytes, the mechanisms by which this signaling pathway directly affects dopamine neurons remain poorly understood. This knowledge gap hinders our comprehensive understanding of the specific role of astrocytes in PD pathogenesis. In this study, the BMP signaling pathway was activated in the “transitional astrocytes” cluster (cluster 4), which showed increased activation of the immune response. BMP signaling is involved in the activation of reactive astrocytes and the formation of glial scars [43, 44], as well as in the regulation of inflammatory responses [45]. In vitro experiments also demonstrated that astrocytes markedly increase the expression of pro-inflammatory cytokines under conditions of PD and BMP signaling activation. Crucially, the inhibition of BMP signaling significantly increased TH expression in the SN and improved motor function in PD model mice. Recent research has demonstrated that extracellular matrix protein-3 (ECM3) binded to extracellular Bmp2 reduces astrocyte-mediated neuroinflammation by inhibiting the BMP signaling pathway, thereby exerting a neuroprotective effect against ischemic stroke [46]. These results underscore the critical role of astrocytes in PD progression and suggest that BMP signaling activation in these cells can exacerbate dopamine neuronal death through the release of inflammatory mediators.

Taken together, the extent and nature of the astrocyte alterations contributed to the pathogenesis of PD. Specifically, the activation of astrocytic BMP signaling drives the release of inflammatory factors, ultimately leading to neuroinflammation and dopaminergic neuron death. Further studies are needed to fully understand their role and to determine whether similar astrocytic changes occur universally across different neurodegenerative diseases. Unraveling the differential activities of astrocytes during disease progression, we aim at enhancing their beneficial effects while mitigating their detrimental effects, thus holding astrocytes as a target for future disease therapy.

## Conclusions

Our findings underscore the crucial role of astrocytes in the pathogenesis of PD. Specifically, we demonstrate that BMP signaling activation in astrocytes drives neuroinflammation, thereby aggravating dopaminergic neuronal loss and motor dysfunction. These findings unveil a novel cellular mechanism underlying PD progression and highlight the potential of targeting astrocytic BMP signaling as a therapeutic strategy for future PD intervention.

## Materials and methods

### Data processing

The expression matrices generated via the cellranger were loaded into the Seurat (v4.0.2) R package for downstream uniform manifold approximation and projection (UMAP) analysis. Cells with 20% mitochondrial genes were excluded from the analysis. Clusters were defined based on the “FindClusters” function with a resolution equal to 0.5. We performed clustering analysis and obtained 25 clusters. The “FindAllMarkers” function was subsequently employed to identify marker genes for each cluster. The marker genes were ranked based on their *p*-value and log2-fold change (log2 FC) within each cluster. Furthermore, we annotated the cell types of the clusters by searching for top-ranked genes in the Cellmarker and Panglao DB databases. After the initial clustering analysis, we obtained the major cell types. We subsequently performed subclustering of the astrocytes with a resolution of 0.2 and merged the results from the two rotations to obtain the final cluster results. For the final dimension reduction and visualization, we employed UMAP.

We used the “FindMarkers” function to identify differentially expressed genes (DEGs) between the PD and CON groups within each cell type. We subsequently applied thresholds of *p* < 0.05 and |log2 FC| > 0.5 to filter the DEGs and obtained PD up- and downregulated genes compared with the control for each cluster. To analyze the functional enrichment of these DEGs, we utilized the R package ClusterProfile (v3.18.4), employing a hypergeometric test to assess enrichment in Gene Ontology (GO) and Kyoto Encyclopedia of Genes and Genomes (KEGG) pathways. Multiple hypothesis testing was corrected via the false discovery rate (FDR).

### Analysis of the whole mouse SN dataset

#### Pseudotemporal trajectory reconstruction

To capture the cellular state changes between the PD group and the CON group within astrocytes, we employed the standard Monocle2 algorithm to reconstruct the cellular state trajectory after subclustering of astrocytes. Identification of trajectory-variable genes that may impact on the cellular state changes between the PD and CON groups.

#### Cell–cell communication analysis

To further investigate the changes in communication between astrocytes in the SN and other cells induced by MPTP, we used the R statistical environment CellChat (v1.4.0) to compute the intercellular communication network. We separately predicted the communication networks for the PD and CON groups’ samples, including information on signaling pathways and ligand-receptor (L-R) pairs, and compared the differences between these two networks.

Interaction count and strength are key factors, so we used the “compareInteractions” function to obtain the differences in interaction count and strength for the entire network. We ranked the conserved signaling pathways based on their Euclidean distance in a shared two-dimensional space. We also compared the information flow of each signaling pathway, which is the sum of the communication probabilities between all the cell pairs, to identify different states of signaling pathways, including closed/open, decreased, and increased.

Finally, we zoomed in to the level of L-R pairs and computed their functional dysregulation through differential expression analysis via the “identifyOverExpressedGenes” and “netMappingDEG” functions. Both upregulated and downregulated L-R pairs were detected. All the plotting functions were implemented via the CellChat package. (based on R software version 4.2.0).

### Establishment of PD model mice

To establish an animal model of MPTP-induced PD, 8-week-old male C57BL/6 mice were treated with 25 mg of 1-Methyl-4-phenyl-1,2,3,6-tetrahydropyridine (MPTP) (cat#: M832929, Macklin) per kg of body weight daily for 5 days (PD group). Moreover, the mice in the CON group were treated with the same dose of physiological saline (CON group). To compare the exercise capacity of the two groups of mice, we performed behavioral testing. All animal experiments complied with the regulations of the Animal Welfare Act of the National Institutes of Health Guide for the Care and Use of Laboratory Animals (NIH Publication No. 85–23, revised 1996) and were approved by the ethics committee of Hebei Medical University (IACUC-Hebmu-P-2023157). Animals with pre-existing health conditions were excluded from the study. All the investigators were blinded. The study was not preregistered.

### Immunofluorescence staining

Frozen sections of mouse SN (10 μm) were removed from the freezer and allowed to return to room temperature. After washing with 0.01 M PBS (cat#: G0002, Servicebio), the mouse SN sections on coverslips were blocked with PBS-T (0.5% Triton X-100) containing 5% donkey serum for 60 min at room temperature (donkey serum was chosen according to the source of the secondary antibody), followed by incubation with primary antibodies diluted in 0.01 M PBS overnight at 4 °C, anti-phospho-SMAD1 (Ser463/465)/SMAD5 (Ser463/465)/SMAD9 (Ser465/467) rabbit mAbs (*p*-Smad1/5/9) (1:2000, cat#: 13820, CST), and anti-Aldh1l1 mouse mAbs (1:500, cat#: 68018-1-Ig, Proteintech). After washing, the coverslips were incubated with the secondary antibodies donkey anti-rabbit IgG (H + L) Highly CrossAdsorbed secondary antibody Alexa Fluor® 594 (1:500, cat#: A-21207, Thermo Scientific™) or donkey anti-mouse IgG (H + L) Highly CrossAdsorbed secondary antibody Alexa Fluor® 488 (1:500, cat#: A-21202, Thermo Scientific™) for 2 h at room temperature. The sections were mounted with DAPI-containing mounting media before imaging. Images were acquired with an inverse Olympus FV1200 microscope equipped with the corresponding fluorescence system and analyzed via ImageJ to determine optical densities for quantitative analysis.

### Immunohistochemical staining

Paraffin sections of mouse SN (5 μm) were dehydrated in different concentrations of ethanol solutions and subjected to antigen retrieval in citrate buffer for immunohistochemical staining. The sections were processed following the instructions provided by the manufacturer via the UltraSensitiveSP Kit (Cat#: KIT-9720, MXB Biotechnologies). To block endogenous peroxidase activity, the sections were treated with 0.3% H_2_O_2_ and then blocked with FBS at 37 °C for 30 min. The sections were subsequently incubated overnight at 4 °C with an anti-TH rabbit mAb (1:200, cat#: PB9449, Boster) and an anti-TH rabbit mAb (1:5000, cat#: A5079, ABclonal). Image analysis and processing were carried out via Image-Pro Plus 6.0 software.

### Cell culture

Spontaneously immortalized Astrocyte type I clone (C8-D1A) cells (cat#: CL-0506, Wuhan Pricella Biotechnology Co., Ltd) were maintained in high-glucose Dulbecco’s modified Eagle’s medium (DMEM) (cat#: C11995500BT, Gibco) supplemented with 10% fetal bovine serum (FBS, cat#: FB15015, clerk) and a 1% penicillin/streptomycin solution (cat#: P1400, Solarbio) in an H_2_O saturated 5% CO_2_ atmosphere at 37 °C. The MN9D cell line (cat#: CL0466, Hunan Fenghui Biotechnology Co., Ltd) was cultured in MEM + NEAA supplemented (cat#: 11095080, Gibco) with 10% FBS and 1% antibiotics (penicillin/streptomycin) in an H_2_O-saturated 5% CO_2_ atmosphere at 37 °C. The cells were digested with trypsin-EDTA (0.25%) without phenol red (cat#: T1300, Solarbio) at 37 °C from 10 cm culture dishes at 70–80% confluence. The cells were then seeded at 50,000 cells/cm^2^ and cultured for another day in 6-well plates (cat#: CCP-6H, Servicebio) for protein/mRNA assays. Both cell lines were recently authenticated by STR profiling and tested for mycoplasma contamination, with all results being negative.

### CCK8 assay

Cell viability was assessed via the CCK-8 assay (cat#: K1018; APEXBIO). The cells (5 × 10^3^ cells/well) were seeded into a 96-well plate and cultured for 24 h. Afterward, the cells were treated with 1-methyl-4-phenylpyridinium (MPP^+^) (or conditioned medium) for various durations: 6, 12, 24, and 48 h. After the treatment, 10 µL of CCK-8 reagent was added to each well, and the cells were incubated for 1 h. The absorbance (OD values) was measured at 450 nm via a microplate reader. The experiment was repeated three times, with three replicate wells for each condition. The data were analyzed via SPSS, and significant differences between the treatment groups were compared. A *p*-value of less than 0.05 was considered statistically significant.

### RT-qPCR

To detect changes in mRNA expression in astrocytes under Bmp6 recombinant protein intervention or in the mouse SN, total RNA was extracted via the Total RNApure Reagent Kit (Cat#: ZP401, ZOMANBIO). First-strand cDNA was generated from 1 μg of total RNA via HiScript III RT SuperMix for qPCR (cat#: R323, Vazyme) with the following three-step incubation: 42 °C for 2 min, 37 °C for 15 min, and 85 °C for 5 s. RT-qPCR was performed via ChamQ Universal SYBR qPCR Master Mix (cat#: KT201, Vazyme). GAPDH was used for normalization. The samples were incubated at 95 °C for 15 min, followed by 40 cycles of denaturation at 95 °C for 10 s, annealing at 60 °C for 30 s, and cDNA extension at 72 °C for 20 s. After the amplification cycles, a final extension step at 72 °C was performed for 10 min. All the experiments were performed in triplicate. The threshold cycle (Ct value) was recorded by a QuantStudio™ 6 Flex Real-Time PCR System (REF#: 4484642, Applied Biosystems). The supplementary materials contain the sequences of primers used in this study. Two-tailed t tests (2 groups) were used for the statistical analyses, and the relative expression of RNAs was analyzed via the 2^−ΔΔ^Ct method. Table S1 lists the primers used for qRT-PCR.

### Western blot

For the preparation of protein samples, astrocytes subjected to Bmp6 recombinant protein intervention or mouse SN tissue were homogenized or lysed in RIPA buffer (cat#: R0020, Solarbio) supplemented with PMSF (100 mM) (cat#: P0100, Solarbio). After ultrasonication and incubation on ice for an hour, the protein samples were cleared by centrifugation at 12,000 × *g* for 20 min at 4 °C, and the supernatant was collected. The protein concentration was evaluated via a BCA protein assay reagent kit (cat#: PC0020, Solarbio) and an Infinite F200 (TECAN, Switzerland) plate reader. Protein samples were mixed with 5× loading buffer (cat#: P1040, Solarbio). After boiling, 20 μg of protein was separated on a 10% SDS polyacrylamide gel and electrotransferred onto polyvinylidene fluoride membranes. The membranes were then blocked with 5% milk in TBST (0.1% Tween-20) for 1 h and incubated with the following antibodies: anti-phospho-Smad1/5/9 (1:1000, cat#: 13820, CST), anti-GAPDH mouse mAb (1:5000, cat#: GB120025, Servicebio), and anti-TH rabbit mAb (1:2000, cat#: A5079, ABclonal). HRP-conjugated AffiniPure goat anti-rabbit IgG (H + L) (1:5000, cat #: BF03008, Biodragon) and HRP-conjugated AffiniPure goat anti-mouse IgG (H + L) (1:5000, cat #: SA00001-1, Proteintech), and an Enhanced/Super ECL Kit (1:1, cat #: BF06053-100, Biodragon) were used. Two-tailed *t* tests (2 groups) or one-way ANOVA (3 groups) were used for the statistical analyses.

### ELISA

After each MPP^+^ treatment or siRNA transfection, the cell culture supernatants were collected for enzyme-linked immunosorbent assay (ELISA). The release of inflammatory cytokines in the cell culture supernatants was measured via the following kits: the Mouse TNF-α ELISA Kit (cat#: EK282, LIANKEBIO), the Mouse IL-1β ELISA Kit (cat#: EK201B, LIANKEBIO), and the Mouse IL-6 ELISA Kit (cat#: EK206, LIANKEBIO).

### Animal behavioral experiments

#### Rotarod experiment

The rotarod test was conducted with a mouse rotarod fatigue tester. The procedure follows: First, the mouse is placed on the rotarod, with an adjustable rotation speed set at 25 rpm. Once the mouse starts crawling, the timer automatically starts recording the time from placement to when the mouse falls off the rod (latency to fall). To ensure reliable results, a non-continuous multiple measurement approach is employed, with one measurement taken per day. Changes in motor ability can be assessed by measuring the duration of a mouse walking on the rotarod.

#### Climbing pole experiment

The mice are placed on a wooden ball with a rough surface at its lower end, which is connected to a cylindrical wooden pole with a rough surface and a circular cross-section. During testing, a mouse was placed on a rough wooden ball with its head pointed upward, and the ability of the animal to climb down to the bottom of the pole without falling through a turn around was used to assess motor function or dysfunction. The lower end of the pole was positioned inside the mouse cage. We recorded the time after the mice were placed, and the time when the head changed from top to bottom was recorded as the head rotation time. When the mouse climbed from the wooden ball onto the wooden pole with its head facing downward, the time was recorded as T1 using a stopwatch. When the mouse reached the lowest point of the pole, the time was recorded as T2. The total time taken by the mouse to climb the entire pole is then calculated via the formula T = T1 − T2. Each mouse underwent two trials, and the average of the two climbing times was used as the statistical indicator.

#### Gait analysis

The gait measurements were collected via an automatic gait analysis system. The mice were placed at one end of the runway and allowed to move to the other end, where they had access to a dark enclosure. Each mouse was housed twice before the formal experiment. The measurement range included the stride length and step speed. The investigator was blinded to the treatment group during behavioral testing.

### Statistical analysis

The data are expressed as the means ± SEMs. Statistical comparisons between two groups were performed via Student’s *t* test. The data met the assumptions of normality (Shapiro–Wilk test, *p* > 0.05) and homogeneity of variance (Levene’s test, *p* > 0.05). For comparisons between multiple groups, one-way ANOVA was conducted, followed by either Fisher’s least significant difference (LSD) test (when equal variances were assumed) or Dunnett’s T3 post hoc test (when variances were unequal), depending on the results of Levene’s test for homogeneity of variances. All the statistical analyses were performed via SPSS 26. Data visualization was carried out via GraphPad Prism 8.0.2. The sample size was selected based on previous experience in the literature, and no formal efficacy analysis was performed.

## Supplementary information

Table S1

Supplementary Figure 1.

Supplementary Figure 2.

Supplementary material legends

Original Western blots

## Data Availability

Full and uncropped western blots are provided as Supplemental Material (Supplementary Fig. 3). The datasets or code used during the current study are available from the corresponding author on reasonable request. The human midbrain scRNA-seq data were derived from the GSE157783 dataset.
