# Supplementary figures and images for: Inhibition of astrocyte BMP signaling alleviates neuroinflammation in experimental models of Parkinson’s disease
Source: Cell Death Discov. 2025 Nov 10;11:528. doi: 10.1038/s41420-025-02812-2 (PMC12603212; doi:10.1038/s41420-025-02812-2)

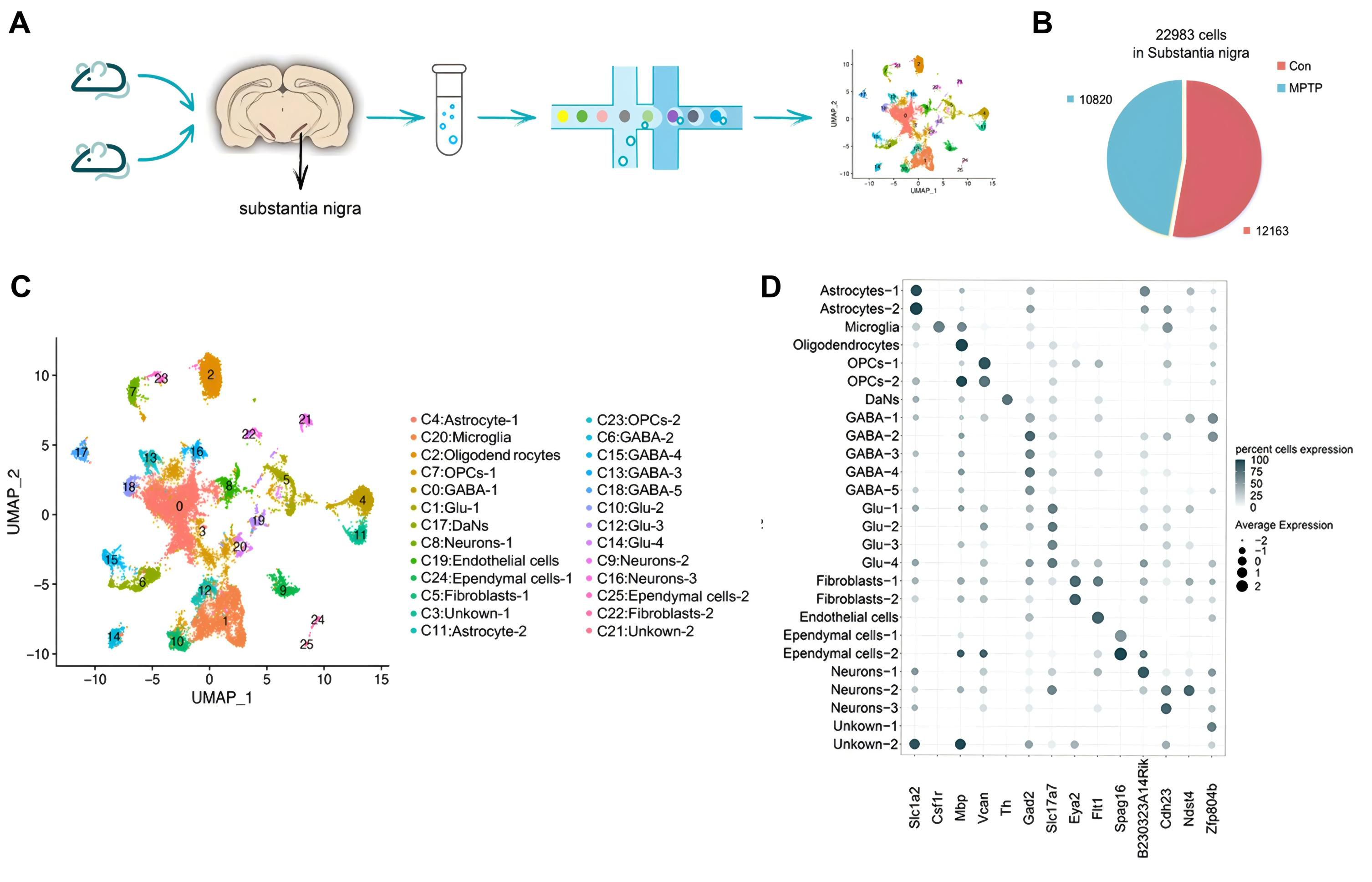

Supplement: Supplementary file 2 — Supplementary Figure 1. [file 41420_2025_2812_MOESM2_ESM.tif]

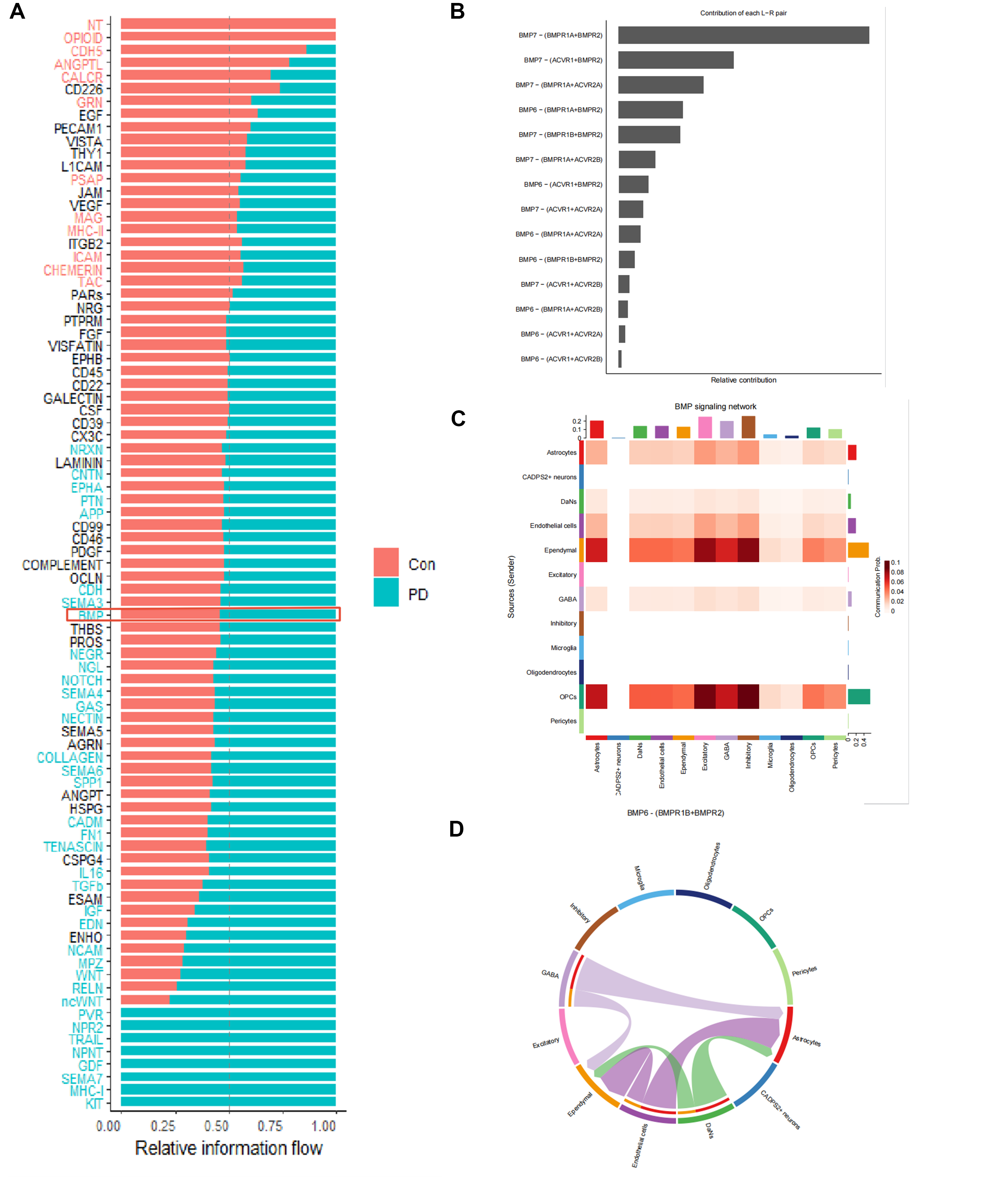

Supplement: Supplementary file 3 — Supplementary Figure 2. [file 41420_2025_2812_MOESM3_ESM.tif]

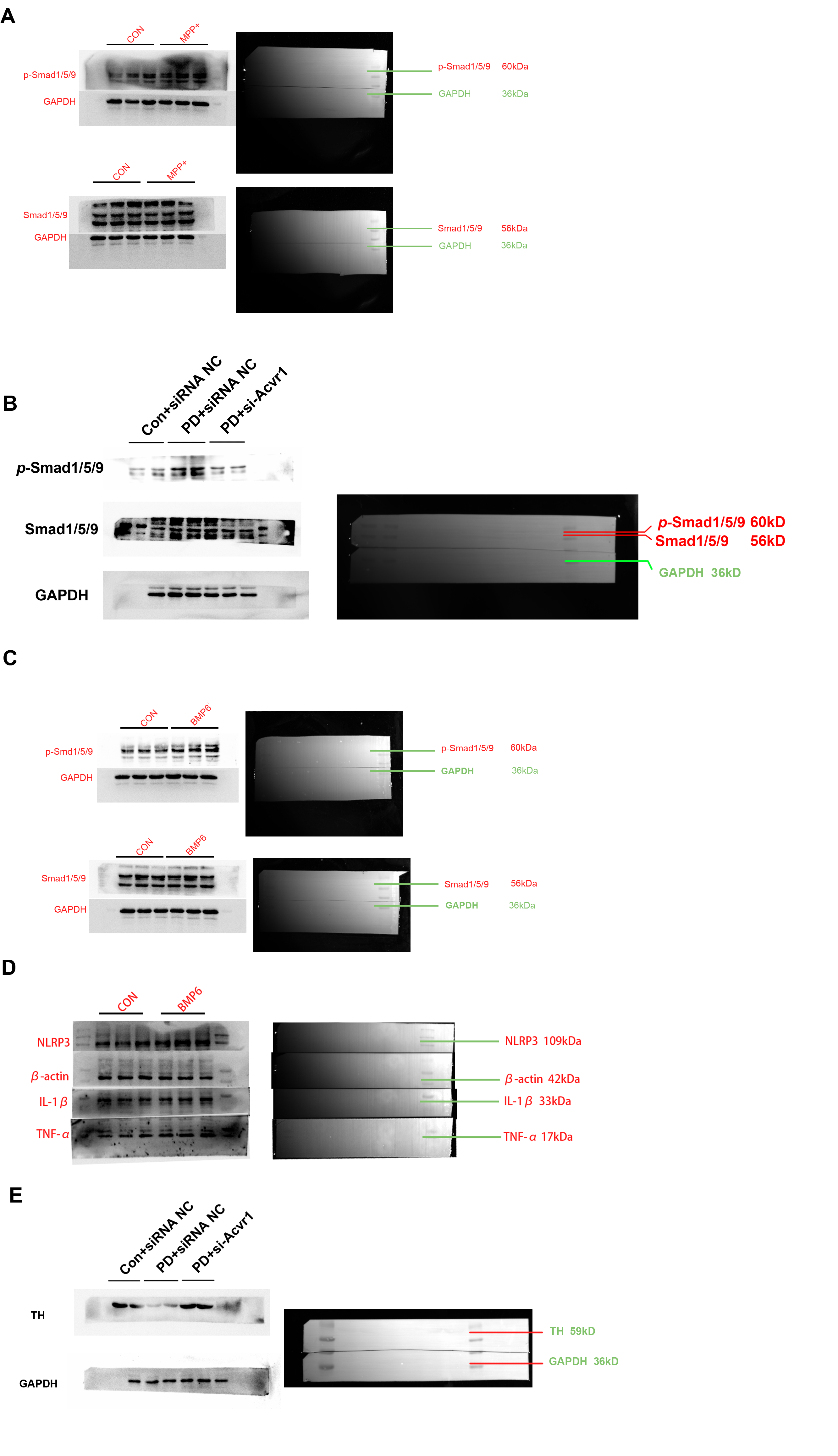

Supplement: Supplementary file 5 — Original Western blots [file 41420_2025_2812_MOESM5_ESM.tif]
