# Supplementary material for: Inhibition of astrocyte BMP signaling alleviates neuroinflammation in experimental models of Parkinson’s disease
Source: Cell Death Discov. 2025 Nov 10;11:528. doi: 10.1038/s41420-025-02812-2 (PMC12603212; doi:10.1038/s41420-025-02812-2)
Supplement: Supplementary file 4 — Supplementary material legends [file 41420_2025_2812_MOESM4_ESM.docx]

**Supplementary Figure 1. Cell type composition of the mouse SN.**

**The figure shows previously published data from our research group.** (A) Experimental approach for SN tissue processing and nucleus extraction. Nuclei suspensions were processed with the 10 × Genomics platform and sequenced with an Illumina sequencer. (B) The number of high-quality nuclei per sample. Overall, the sample consisted of 10,820 nuclei from the MPTP group and 12,163 nuclei from the control group. (C) UMAP embedding of the 22,983 mouse SN nuclei; colored by cluster. (D) Cell representative marker genes. Expression level (color scale) of marker genes across clusters and the percentage of cell expression (dot size).

**Supplementary Figure 2. BMP signaling pathway activation in human midbrain scRNA-seq data.** (A) Signaling pathways were ranked by differential information flow (total communication probability) between PD and Con networks. (B) Relative contribution of each L-R pair to the overall BMP signaling network at PD. (C) Heatmap of the interactions between the BMP signaling pathway sending and receiving cells. (D) Chord diagram of the inferred BMP signaling network received by astrocytes.
